# Determination of Cassiarin A Level of *Cassia siamea* Leaf Obtained from Various Regions in Indonesia Using the TLC-Densitometry Method

**DOI:** 10.1155/2020/7367836

**Published:** 2020-08-07

**Authors:** Wiwied Ekasari, Yuli Widiyastuti, Dyah Subositi, Rini Hamsidi, Aty Widyawaruyanti, Sukmawati Basuki, Dwi Setyawan

**Affiliations:** ^1^Department of Pharmacognosy and Phytochemistry, Faculty of Pharmacy, Universitas Airlangga, Surabaya 60115, Indonesia; ^2^Medicinal Plant and Traditional Medicine Research and Development Center, National Institute Health Research and Development, Ministry of Health, Karanganyar 57792, Indonesia; ^3^Department of Health, Faculty of Vocational Studies, Universitas Airlangga, Surabaya 60115, Indonesia; ^4^Department of Medical Parasitology, Faculty of Medicine, Universitas Airlangga, Surabaya 60131, Indonesia; ^5^Department of Pharmaceutics, Faculty of Pharmacy, Universitas Airlangga, Surabaya 60115, Indonesia

## Abstract

*Cassia siamea* leaf has been proven *in vitro* and *in vivo* to have a strong antimalarial activity with Cassiarin A as its active compound. To obtain a source of *C. siamea* medicinal plant with high level of active antimalarial compound (Cassiarin A), a valid method for determining Cassiarin A level is needed. For this reason, this research conducts the validation of the Cassiarin A content with determination method using thin-layer chromatography (TLC) densitometry which includes the determination of selectivity (*R*s), linearity (r), accuracy, precision, limit of detection (LOD), and limit of quantification (LOQ). Cassiarin A was chromatographed on silica gel 60 F_254_ TLC plate using chloroform : ethanol (85 : 15 v/v) as a mobile phase. Cassiarin A was quantified by densitometric analysis at 368 nm. The linear regression analysis data for the calibration plots showed good linear relationship with *r* = 0.9995. The method was validated for precision, recovery, repeatability. The minimum detectable amount was found to be 0.0027 *μ*g/spot, whereas the limit of quantitation was found to be 0.008 *μ*g/spot. The results of this validation are then used to determine the Cassiarin A level of *C. siamea* leaf from various regions in Indonesia. Based on the results of the study, it can be concluded that the TLC-densitometry method can be used to determine level of the Cassiarin A compound with the advantages of being fast, easy, accurate, and inexpensive. In addition, it showed that *C. siamea* leaves from Pacitan have the highest level of Cassiarin A compared to other areas studied.

## 1. Introduction

*Cassia siamea* L. is one of the plants in Indonesia that has traditionally been recognized for its ability to treat malaria. This plant grows better in the lowlands, with low to high rainfall (optimum around 1000 mm), an average temperature of 20°C–31°C, and with a four to eight months dry season, and spreads to Java at an altitude of less than 1000 masl [1]. Various *in vitro* as well as *in vivo* studies have been carried out in some plant leaves of the *Cassia* genus of Caesalpiniaceae family for its activity as an antimalarial with *C. siamea* leaf yield shows the highest antimalarial activity of other plants of the *Cassia* genus [2–4].

**Figure 1:**
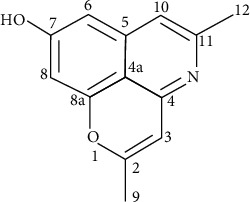
Structure of Cassiarin A.

Other *in vitro* and *in vivo* antimalarial studies on *C. siamea* leaf which in Indonesia is known by the name *Johar* successfully obtain new active antimalarial compounds namely Cassiarin A and Cassiarin B with the highest antimalarial activity possessed by Cassiarin A compound (see Figure 1) [5].

It is known that the levels of compounds in medicinal plants are influenced by many factors, including the place of growth, harvest time, how to cultivate, postharvesting processing, and so on [6]. Plants of the same species growing in different environments can have differences in secondary metabolites in certain concentrations [7]. This is influenced by several environmental factors including biotic and abiotic stimuli that regulate biosynthesis of plant secondary metabolites [8]. Several studies have reported that locations or regions with different latitudes, longitudes, average temperatures, and climatic conditions affect the production of secondary plant metabolites and have an effect on their biological activity [9–13].

Until now, there has been no research to quantitatively determine Cassiarin A compound in *C. siamea* leaf. To get a source of medicinal plants with high levels of active antimalarial compounds (Cassiarin A), then a valid method for determining levels of Cassiarin A is needed. Quantification of Cassiarin A as an antimalarial active compound in *C. siamea* leaf using thin-layer chromatography (TLC) densitometry to date has never been reported.

TLC techniques is commonly utilized by the pharmaceutical industry in the process of developing, identifying, and detecting counterfeiting in herbal products and assisting in the identification of mycotoxin content and pesticides and in the quality control of herbs and health foods, including validation of herbal treatment methods [14]. The detector that is on the densitometer allows TLC to achieve the accurate qualitative and quantitative results. One example of the use of TLC-densitometry is for the determination of phyllanthin and gallic acid in herbal hepatoprotective formulations [15].

Thin-layer chromatography (TLC) is a rapid screening method for separating compounds and identifying herbal extracts. This method is often used as a qualitative and quantitative analysis with accurate, precise, and reliable procedures, relatively low operating costs, and short time for analysis, and it is easy to use [16]. TLC has the unique advantages over other chromatographic techniques such as HPLC, GC, and CE which have higher separation and selectivity capabilities. These advantages are as follows: (1) the disposable property of TLC can avoid the cross contamination, especially for all substances which are adsorbed to the stationary phase of column chromatography and can reduce the life of the column and produce a bad peak shape, (2) relatively, a short time is needed to train operators due to the easy TLC operation, and (3) finally, the visible light as well as sensitive visualization reagents can be easily used to identify and characterize almost all compounds [17].

Thus, the aim of this study is to develop and validate the TLC-densitometry method to determine Cassiarin A content and determine Cassiarin A content in *C. siamea* leaf collected from various different locations in Indonesia.

## 2. Materials and Methods

### 2.1. *Cassia siamea* Leaf

*C. siamea* leaf is collected from 17 different locations in Indonesia and has been identified by Anshari Maruzi from Systematic Laboratory, with a voucher specimen number YK.01.03/2/2861/2019 and deposited in the Herbarium Tawangmanguense at Medicinal Plant and Traditional Medicine Research and Development Centre, Ministry of Health Republic of Indonesia.

### 2.2. Cassiarin A

Cassiarin A standard compound was obtained from the synthesis results by Dr. Marcellino Rudyanto from the Faculty of Pharmacy, Universitas Airlangga, Surabaya [18].

### 2.3. Standard Solution Preparation

One mg of standard Cassiarin A was dissolved in 1 mL of chloroform-ethanol (85 : 15 v/v) mixture and diluted to obtain a 50–700 ppm dilution series of standard solution and then stored in a refrigerator at 4°C.

## 3. Method Validation

TLC-densitometry from the content of Cassiarin A in the *C. siamea* leaf ethyl acetate fraction validated in terms of determining wavelength, selectivity, linearity, accuracy, precision, limit of detection (LOD), and limit of quantification (LOQ) according to The United States Pharmacopeia (USP) [19].

The maximum wavelength was determined by analysis of three standard concentrations of Cassiarin A in the wavelength range of 200–370 nm. The selected maximum wavelength would be used for determination of content. Linearity was performed by analysis of six standard concentrations of Cassiarin A in the range of 200, 300, 400, 500, 600, and 700 ppm. Linearity was indicated by the value of the correlation coefficient (*r*). Accuracy was stated with percentage recovery which could be calculated as

(1) $$\begin{array}{c} \%\text{ recovery}=\frac{\text{total concentration of samples obtained}}{\text{actual sample concentration }+\text{concentration of analyte added}}\times 100\%. \end{array}$$

The precision was determined on the same day with different bottling quantities and was expressed as a percentage of relative standard deviation (% RSD). Determination of LOD and LOQ was done by bottling a standard solution of Cassiarin A with the concentrations of 200, 50, 25, 10, and 5 ppm as much as 2 *µ*L on the TLC plate. Then, chromatograms of the smallest levels obtained that could be detected by densitometers were up to four kinds of levels above the smallest levels. LOD and LOQ were calculated from the calibration curves of 3.3 (SD/S) and 10 (SD/S), where SD was the standard deviation of the regression line and S was the slope of the regression line.

Selectivity was determined from the eluent optimization results by calculating the resolution (*R*_s_) value. The selective effluent would be used as the chosen mobile phase in determining the content. In addition, the selectivity of this method was ensured by analyzing standard solutions and sample fractions. The Cassiarin A band in the fraction sample was confirmed by comparing the retention (*R*_F_) value and ultraviolet (UV) absorption spectrum against the standard Cassiarin A band. The peak sample purity was assessed by comparing the Cassiarin A standard overlay spectrum and fraction extracts at three different positions, starting at the peak, apex peaks, and peak end position where it was detected at 368 nm.

### 3.1. Preparation of the *C. siamea* Leaf Ethyl Acetate Fraction

Ten mg of *C. siamea* leaf powder was extracted with n-hexane using the sonication extraction method, and then the solid residue was extracted with 90% ethanol containing 1% tartaric acid. Macerate obtained was made into base with ammonium hydroxide (NH_4_OH) to pH 8, allowed to stand for 24 hours, and then evaporated to dry. The dried extract obtained was then dissolved with distilled water and withdrawn with ethyl acetate. The ethyl acetate phase obtained was then stored for analysis using the TLC-densitometry method.

### 3.2. TLC-Densitometry Method

Twenty microliters of 17 *C. siamea* leaf ethyl acetate fractions and the standard solution of Cassiarin A were bottled on a *silica gel* 60 GF_254_ 20 × 10 cm plate (E. Merck, Germany) using CAMAG® Linomat 5 (Camag, Switzerland) under the flow of nitrogen gas. Each sample was bottled in the form of a ribbon with a length of 4 mm with a distance between the bands that was 4.5 mm. The TLC plate was eluted inside CAMAG® glass (20 × 10 cm) chamber, which had been saturated with a mobile phase of chloroform and ethanol (85 : 15 v/v) for 70 minutes at room temperature. The plate was scanned below the wavelength of 368 nm using CAMAG® TLC Scanner 3 (CAMAG®, Switzerland) with winCATS software. Cassiarin A level in the ethyl acetate fraction of *C. siamea* leaf was calculated based on the peak area. The test was repeated three times.

## 4. Results and Discussion

**Figure 2:**
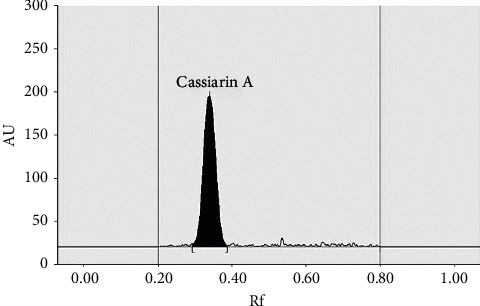
Single chromatographic band of Cassiarin A indicating its purity.

Cassiarin A as an antimalarial bioactive compound from the *C. siamea* leaf was used as a marker compound in this study. An analytical method for determining Cassiarin A level from the ethyl acetate fraction of *C. siamea* leaf from various regions was done using TLC-densitometry. Chromatographic condition for measuring Cassiarin A level was done using silica gel 60 GF_254_ plate. The mobile phase chosen was chloroform and ethanol (85 : 15 v/v) showing the best separation of Cassiarin A in the *C. siamea* L. leaf ethyl acetate fraction with a *R*_F_ value of 0.34 at a wavelength of 368 nm. The Cassiarin A stain from the fraction sample was confirmed by comparing the *R*_F_ value against the Cassiarin A standard (see Table 1 and Figure 2).

According to USP, analytic method is validated to confirm that analytical procedure uses reliable and accurate data. This method is validated for selectivity, linearity, accuracy, precision, LOD, and LOQ. Selectivity was confirmed by comparing the UV spectrum of all 17 samples with the Cassiarin A standard. The results showed maximum absorbance at a wavelength of 368 nm. In addition, from the experimental results of three kinds of mobile phase systems, the chloroform–ethanol mobile phase (85 : 15 v/v) showed the best Cassiarin A separation with a *R*_s_ value of 2.20. This value meets the requirements where a good resolution is > 1.5 [20]. Good correlation was also obtained from spectrum overlay between Cassiarin A standards and Cassiarin A in fraction sample with a correlation value of 0.969.

The calibration curve for Cassiarin A level in the *C. siamea* leaf ethyl acetate fraction using the TLC-densitometry method showed a good linearity relationship in the range of 0.40–1.40 *μ*g/spot with a correlation coefficient (*r*) of more than 0.99.

Recovery value of TLC-densitometry method of *C. siamea* leaf ethyl acetate fraction was still within the acceptable limit, which was 102.81–120.14%. Accuracy was determined by calculating the percentage of standard recovery added to each sample that was 80%, 100%, and 120% of the expected samples concentration. Almost all publications were complied with the number of replicates (*n* ≥ 3) at each of the concentration levels studied [21]. The average of percentage recovery value and RSD value obtained from this study were 102.81 ± 1.45 and 1.42%; 108.13 ± 2.71 and 2.51%; 120.14 ± 4.75 and 3.95%. The precision of this method is less than 5% RSD, in which it meets the requirements of precision of natural materials in general.

LOD and LOQ for TLC-densitometry method of *C. siamea* leaf ethyl acetate fraction at the wavelength of 368 nm were each 0.0027 and 0.0080 *μ*g/spot. Based on these results, it is known that, to get good accuracy and precision, it must work at levels above 0.0080 *µ*g. The TLC-densitometry validity of the *C. siamea* leaf ethyl acetate fraction is presented in Table 2.

The results of *C. siamea* leaf ethyl acetate fraction from various regions by sonication extraction and determination of Cassiarin A level using TLC-densitometry are presented in Table 3.

**Figure 4:**
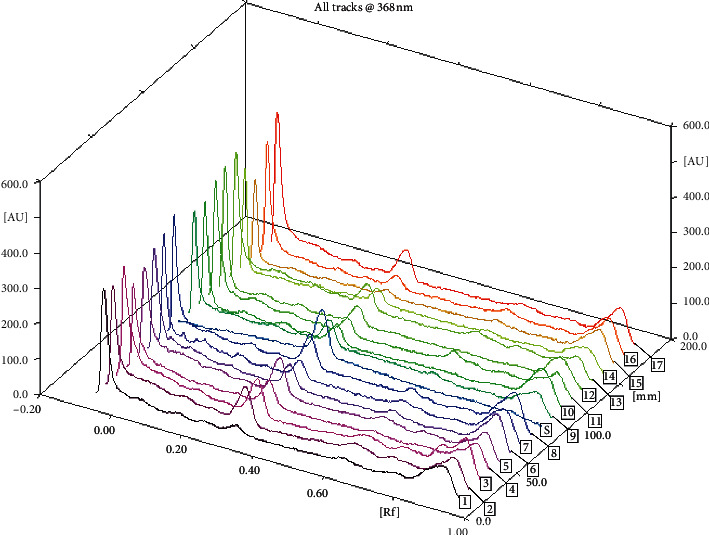
Chromatogram profile of Cassiarin A standard S and samples (numbering as in Table 3).

The chromatogram profile of Cassiarin A from *C. siamea* scanned at a wavelength of 368 nm was shown in Figure 4. The highest Cassiarin A level was obtained from the sample from Pacitan with level of 0.0117% ± 0.0004, while the lowest Cassiarin A level was obtained from the sample from Surabaya with level of 0.0014% ± 0.0001.

Based on the geographical data, Pacitan district is located at an altitude of 36 m above sea level (asl) [22], which has a type of red Mediterranean litosol, alluvial gray clay deposits, and reddish litosol complexes. Average monthly rainfall ranges from 4.48 to 23.80 mm. The air temperature in Pacitan district is in the range of 24–26°C with an average humidity of 96% [23], while Surabaya is located at 2 m above sea level (asl) and has an average rainfall of 172 mm with temperatures around at 30°C, humidity of 68%–84%, and soil type of mostly alluvial soils (soil formed from sedimentary river mud) [24].

Some studies have showed that different environmental conditions caused differences in the content of Cassiarin A. Hendrison et al. (2001) reported that differences in altitude caused the different secondary metabolite content [25]. A research conducted by Figueiredo et al. (2008) explained that the metabolite content in plants was influenced by many factors, including genetic and environmental (biotic and abiotic) factors, physiological conditions, geographical variations, and evolution [26].

This research is the first study about the validation of Cassiarin A level determination as an active antimalarial compound of *C. siamea* leaf using TLC-densitometry. Other studies on quantitative analysis with TLC-densitometry have been carried out on the leaves and flowers of this plant to determine the level of barracol [27]. The data obtained from this study are very valuable to show the source of *C. siamea* plant with the desired Cassiarin A content. Validation of determination of Cassiarin A level with TLC-densitometry is very useful also in controlling the quality of herbal medicinal ingredients from *C. siamea* leaf to ensure the consistency of safety and effectiveness of herbal products, especially as an antimalarial drug. The European Medicines Agency (EMA) defines marker compound as the compound that exists in medicinal plants that is used for the matter of quality control regardless of whether it has a therapeutic effect or not. EMA categorizes marker compounds in two categories, namely, analytic marker and active marker [6, 28]. Thus, Cassiarin A can be used as an active marker compound from *C. siamea* plant leaf as an antimalarial. The study of marker compound can also be applied to the process of ascertaining the authenticity of species, finding new sources or replacing raw materials, optimization of extraction methods, purification, elucidation of structures, and determination of purity [28].

## 5. Conclusions

Based on the results of the study, it can be concluded that the TLC-densitometry method can be used to determine levels of Cassiarin A compound with several advantages such as fast, easy, accurate, and inexpensive. In addition, *C. siamea* leaf from Pacitan has the highest level of Cassiarin A compared to other regions.

## Figures and Tables

**Figure 3:**
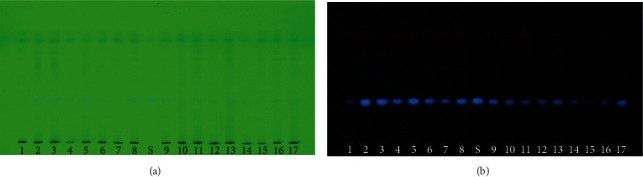
Cassiarin A standard (S) and *C. siamea* leaf ethyl acetate fraction (numbering as in Table 3) from 17 regions in Indonesia. (a) TLC plate under UV 254 nm. (b) TLC plate under UV 366 nm.

**Table 1:** Characteristics of Cassiarin A [18].

Method		Characteristic
1	Molecular weight	213.23 g/mol
2	*R*_F_ value	0.34 ± 0.01 (silica GF_254_, chloroform : ethanol 85 : 15)
3	Color	Reddish solid
4	UV	*λ*_max_ (EtOH) at 368 nm
5	IR	3420 cm^−1^ and 2940 cm^−1^ (characteristic peak of hydroxyl group (-OH) and/or (NH))
		1660 cm^−1^ and 1620 cm^−1^ (characteristic peak of ether)
		1395 cm^−1^, 1370 cm^−1^, and 1190 cm^−1^
6	^1^H-NMR position (numbering as in Figure 1)	Chemical shift (ppm)
	3	6.03 (1H, *s*)
	6	6.46 (1H, *s*)
	8	6.48 (1H, *s*)
	9	2.20 (3H, *s*)
	10	6.70 (1H, *s*)
	12	2.34 (3H, *s*)

**Table 2:** Validation of the TLC-densitometry method of Cassiarin A in *C. siamea* leaf ethyl acetate fraction.

Parameters	
Selectivity (*R*_s_)	2.20 (chloroform : ethanol = 85 : 15 v/v)
Linearity (*r*)	0.9928
Accuracy (% recovery)	102.81–120.14
Precision (% RSD)	1.42–3.95
Detection limit (*μ*g/spot)	0.0027
Quantitation limit (*μ*g/spot)	0.0080

**Table 3:** The levels of Cassiarin A in the *C. siamea* leaf from various regions.

No.	Sources (region in Indonesia)	Cassiarin A levels (% w/v)
1	Tawangmangu, central Java	0.0025% ± 0.0001
2	Ambarawa (east region), Semarang, East Java	0.0082% ± 0.0003
3	Ambarawa (west region), Semarang, East Java	0.0078% ± 0.0003
4	Kediri (north region), East Java	0.0068% ± 0.0003
5	Kediri (south region), East Java	0.0069% ± 0.0003
6	Purworejo, central Java	0.0057% ± 0.0003
7	Gunungkidul, Yogyakarta, central Java	0.0039% ± 0.0003
8	Lembeyan, Magetan, East Java	0.0068% ± 0.0003
9	Pacitan, East Java	0.0117% ± 0.0004
10	Palu, central Sulawesi	0.0062% ± 0.0002
11	Andalas, west Sumatra	0.0036% ± 0.0001
12	Pariaman, west Sumatra	0.0101% ± 0.0010
13	Karanganyar, central Java	0.0056% ± 0.0002
14	Wonogiri, central Java	0.0040% ± 0.0003
15	Surabaya, East Java	0.0014% ± 0.0001
16	Magetan, East Java	0.0041% ± 0.0001
17	Kediri (city), East Java	0.0074% ± 0.0004

TLC chromatograms of *C. siamea* leaf ethyl acetate fraction under UV 254 nm and 366 nm are shown in Figure 3, respectively.

## Data Availability

The data used to support the finding of this study are available from the corresponding author upon request.
